# The effectiveness of specialist cognitive behavioural therapy for functional neurological disorder: a service evaluation

**DOI:** 10.3389/fpsyt.2026.1786295

**Published:** 2026-03-13

**Authors:** Rhiannon White, Susannah Pick, Trudie Chalder

**Affiliations:** 1 Department of Psychological Medicine, Institute of Psychiatry, Psychology & Neuroscience, King’s College London, London, United Kingdom; 2 Division of Psychology and Language Sciences, University College London, London, United Kingdom

**Keywords:** cognitive-behavioural approach, functional symptoms, psychological distress, psychological therapies, routine clinical practice

## Abstract

**Introduction:**

Functional neurological disorder (FND) is a common and disabling condition associated with high levels of functional impairment and psychological distress. Psychological therapies such as cognitive behavioural therapy (CBT) are increasingly recommended as part of multidisciplinary care, but evidence from real-world clinical settings remains limited.

**Methods:**

This retrospective observational cohort study evaluated outcomes from a specialist CBT programme for adults with FND using routinely collected clinical data. Self-report measures of depression, anxiety, psychological distress, functional impairment, physical functioning, pain, and six cognitive-behavioural responses were collected before, during, and at the end of treatment. Linear mixed-effects models were used to examine change over time, with correction for multiple testing.

**Results:**

Data from 234 patients were analyzed (70.5% female; mean age 40.9 years). Despite low completion of measures at follow up, significant improvements were observed in psychological distress, functional impairment, and five of the six cognitive-behavioural response domains across treatment, whereas no significant change was seen in physical functioning or pain measures. Sensitivity analyses excluding patients who received three or fewer sessions produced a consistent pattern of results.

**Conclusions:**

These findings suggest that specialist CBT for FND delivered in routine clinical practice is associated with meaningful improvements in distress, functioning, and key cognitive-behavioural maintenance processes, supporting its role within multidisciplinary care.

## 1 Introduction

Functional neurological disorder (FND) is a clinical syndrome of distressing alterations in sensory, motor or cognitive function, which are proposed to be due to the dysfunction of brain networks as opposed to identifiable structural pathology (1, 2). It is a highly prevalent disorder, representing one of the most common causes of referrals to neurology clinics (3). FND is seen to disproportionately impact women, who make up around 70% of cases (4, 5). The disorder is associated with substantial functional impairment, reductions in quality of life and psychiatric comorbidity (6).

FND is increasingly understood within a biopsychosocial framework, in which symptoms arise from dynamic interactions between neurobiological processes, psychological factors, and social/contextual influences (2, 7). Altered attentional processes are considered central (8), with symptoms becoming more prominent when attention is disproportionately focused on internal sensations or movements, and reduced when attention is diverted or when patients are in automatic action states (9). Difficulties in emotion regulation and heightened affective arousal may contribute to symptom generation, with neuroimaging evidence demonstrating increased limbic reactivity and altered connectivity between emotion-processing and motor networks in FND (7, 10). A higher prevalence of psychosocial adversity and trauma histories has also been observed in FND populations (11), which may contribute to vulnerability for symptom development and ongoing distress (12).

Cognitive-behavioural models propose that once symptoms develop, a range of cognitive and behavioural responses can inadvertently maintain or exacerbate them. Catastrophic interpretations, heightened symptom-focused attention, and avoidance activity patterns can increase distress and functional impairment over time (13, 14). Early case-level evidence provided preliminary support for this model, demonstrating that reductions in avoidance and symptom-focus were associated with sustained recovery from functional seizures following CBT (15). More recent empirical research has strengthened this account (16, 17). Mediation analyses of outcome data for persistent physical symptom populations have shown that changes in unhelpful cognitive and behavioural responses (particularly catastrophising and symptom focusing) can predict improvement in functioning (18). Similarly, mediation analysis of the CODES trial demonstrated that CBT-related improvements in avoidance behaviour and unhelpful cognitions were associated with improvements in functional outcomes and quality of life in patients with dissociative seizures (19).

Cognitive behavioural therapy (CBT) seeks to identify interconnections between emotions, unhelpful thoughts, behaviours and bodily sensations that are thought to perpetuate functional symptoms. Contemporary approaches are increasingly process-focused, targeting transdiagnostic psychological and behavioural mechanisms rather than depending on a fixed treatment protocol (20). This is consistent with the broader movement toward process-based therapy, which emphasises identifying and modifying empirically supported mechanisms of change that cut across diagnostic categories, rather than delivering disorder-specific treatment interventions (21). This is consistent with mechanistic research demonstrating the role of attentional processes, maladaptive beliefs, and avoidance behaviours in the maintenance of functional neurological symptoms (7, 8, 13). CBT for FND aims to reduce symptom-focused attention, challenge maladaptive beliefs, and support graded re-engagement in meaningful activities, thus enabling improvements in coping and daily functioning even where physical symptoms may persist. By helping patients to reframe self-defeating illness beliefs, shift attention away from symptoms, and develop more adaptive strategies for emotional and physiological regulation, CBT may directly address the psychological and physiological processes sustaining functional symptoms. These psychological and behavioural processes represent key mechanisms hypothesised to maintain symptoms and are therefore important targets for therapeutic intervention.

There is emerging evidence for the efficacy of psychological treatments such as CBT in the management of FND. Early feasibility work suggested CBT may reduce functional seizure frequency and improve functioning (22). The CODES trial (23), demonstrated that CBT plus standardised medical care (SMC) was superior to SMC alone at 6 months in significantly reducing monthly seizure frequency for adults with functional seizures. However, this was not sustained at 12 months follow up. Nevertheless, CBT plus SMC did produce improvements across a range of clinically relevant secondary outcomes, such as reduced symptom-related distress, improved health-related quality of life and lower psychological distress at 6 and 12 months. A subsequent mediation analysis suggested that CBT improved avoidance behaviour, anxiety and depression, and that these changes partially mediated improvements in seizure frequency, functional impairment and mental health-related quality of life (19).

Evidence for process-focused CBT approaches also comes from related persistent physical symptom populations. The PRINCE trial (20), conducted in secondary care, evaluated a process-focused CBT intervention targeting transdiagnostic maintaining mechanisms such as avoidance, threat appraisal, and symptom-focused attention. The intervention was associated with improvements in distress and functioning, supporting the relevance of targeting shared psychological processes rather than symptom-specific outcomes alone, and aligning with contemporary CBT approaches used in specialist FND services.

A systematic review of 19 psychotherapy studies for adults with FND (12 of which focused on a CBT intervention) reported moderate to large effects on physical symptoms and small to moderate effects on mental health, functioning and quality of life, with broadly comparable outcomes for CBT and psychodynamic approaches (24). Outcomes were generally similar across CBT and psychodynamic psychotherapy interventions. More recently, a meta-analytic review of 44 psychotherapy studies for functional seizures (25) found that psychological interventions were associated with moderate improvements in seizure outcomes, although most studies were at medium-high risk of bias. In addition, a subsequent multidisciplinary outpatient programme combining CBT, physiotherapy, psychoeducation and self-management has shown benefits for psychological wellbeing and functional performance in patients with FND (26).

Therefore, while previous research has provided some support for the value of CBT in FND management, important gaps remain regarding its effectiveness when delivered within routine specialist clinical services, and the extent to which cognitive-behavioural processes hypothesised to maintain symptoms change in real-world treatment settings. This study used a retrospective observational cohort design to examine the potential effectiveness of specialist CBT for FND within a real-world clinical setting. This was achieved through analysis of routinely collected clinical outcome data collected as part of standard clinical care and extracted for service evaluation purposes, across domains of psychological wellbeing, functional impairment, and cognitive-behavioural responses implicated in symptom maintenance. By evaluating outcomes across the course of treatment, this study seeks to contribute to the growing evidence base for psychological interventions in FND and to inform ongoing service development. Given previous findings that psychological therapies may produce greater improvements in psychological distress and functional impact than in core physical symptoms (23, 26) any changes in physical symptom burden were anticipated to be smaller in magnitude.

## 2 Materials and methods

### 2.1 Participants

Participants were adults referred to the service with a clinical diagnosis of FND made in line with DSM-5 criteria. As a tertiary level service, accepted patients had identified FND with interfacing neurological and mental health symptoms beyond what could be offered to them locally. Patients were primarily working-age adults (18-65), with those over 65 considered on an individual basis. Accepted participants received 1–2 initial assessment sessions before beginning treatment, which focused on clinical assessment and formulation development. If deemed suitable, a course of CBT treatment was offered over 12–14 weeks. Sessions did not follow a fixed, manualized protocol but were flexibly adapted based on each individual patient’s formulation.

All participants who entered the CBT programme and completed at least one outcome measure at any timepoint were eligible for inclusion. Participants were not excluded because of comorbid psychiatric or medical diagnoses. Detailed diagnostic characterisation of comorbidities and information regarding concurrent pharmacological or psychological treatments were not consistently available within the routinely collected dataset and were therefore not included in the present analysis. Nevertheless. we did have self-report measures assessing depressive and anxious symptoms and distress.

### 2.2 Programme structure and delivery

Participants underwent an initial 2-hour assessment, followed by up to 12 one-to-one treatment sessions lasting one hour. These were typically weekly but sometimes spaced out to fortnightly towards the end of treatment. All treatment was delivered in person by one of four specialist CBT therapists. Treatment fidelity was not specifically assessed, however all clinicians received regular clinical supervision by a senior clinical psychologist or specialist psychotherapist.

Sessions followed an adapted CBT for FND structure, in line with previous trials (23). Initial sessions focused on formulation and goal setting. In collaboration with their therapist, patients worked on strategies for managing functional symptoms, behavioural experiments to address avoidances, cognitive strategies to address unhelpful thoughts or misattributions, problem-solving strategies, emotional processing techniques, and relapse prevention towards the end of treatment. Short-term trauma-focused work was incorporated when clinically indicated. Homework tasks (e.g., behavioural experiments, graded exposure, activity scheduling, and cognitive restructuring exercises) were assigned between sessions to support generalisation. Approval for clinical evaluation was given by the South London and Maudsley NHS Foundation Trust (SLAM) Psychological Medicine and Older Adults Directorate.

### 2.3 Outcome measures

Participants were invited to complete a set of self-report questionnaires at three timepoints; pre-treatment, mid-treatment (session 7) and at the end of treatment. These assessed several of the recommended core outcome domains (27). The General Anxiety Disorder-7 (GAD-7) is a valid tool for screening for generalised anxiety disorder (28), whereby a score of 10 out of 21 or more indicates severe anxiety. The Patient Health Questionnaire-9 (PHQ-9) was used to assess the severity of depression (29), with a score of 10 or more out of 27 indicating moderate depression. The Clinical Outcomes in Routine Evaluation (CORE-10) is a tool measuring psychological distress (30) that includes anxiety, depression, daily functioning and risk to self.

The RAND-36 is a measure that taps eight health-related quality-of-life concepts (31), from which the physical functioning and pain subscales were used. The Work and Social Adjustment Scale (WSAS) provided a further, reliable and valid measure of physical functioning (32), in which a score of 10/40 or more indicated moderate functional impairment.

The Cognitive Behavioural Responses to Symptoms Questionnaire (CBRQ) was used to assess cognitive and behavioural processes that may be implicated in FND: Symptom Focusing, Embarrassment Avoidance, Fear Avoidance, Damage Beliefs, All or Nothing Behaviour, Avoidance/Resting Behaviour (33, 34).

### 2.4 Data analysis

Participants’ demographics and patient-reported outcome measures were summarised descriptively. To examine change over time, linear mixed-effects models with random intercepts for participants were used to enable inclusion of all available data, including from participants with incomplete follow-up. Time (Pre, Mid, End) was entered as a fixed effect, and a diagonal residual covariance structure was specified. Pairwise contrasts between timepoints were examined using least significant difference (LSD) tests. Effect magnitude is presented as estimated mean differences with 95% confidence intervals derived from the mixed-effects models.

Given that twelve primary outcome models were estimated, the Benjamini–Hochberg false discovery rate (FDR) procedure (5%) was applied to control for multiple comparisons. After FDR correction, nine of the twelve outcomes showed a significant effect of time.

## 3 Results

A total of 234 individual participants contributed data from at least one time point. A summary of participant demographics and FND diagnostic type can be seen in **Table 1**.

**Table 1:** Summary of patient demographic information.

Characteristic	Category	N	%
Gender	Female	165	70.5%
	Male	54	23.1%
	Not reported	15	6.4%
Ethnicity	White British	115	49.1%
	Not reported	54	23.1%
	Black British	19	8.1%
	White Other	10	4.3%
	English	9	3.8%
	Asian/Asian British	8	3.4%
	Mixed Race/Mixed Race British	6	2.6%
	African	6	2.6%
	Nigerian	2	0.9%
	European	2	0.9%
	Turkish	2	0.9%
	Irish	1	0.4%
Age	Mean (range)	40.9 (19–78)	–
		N	Mean (SD)
Clinical characteristics	Duration of symptoms (years)	–	8.84 (9.11)
	Participants reporting seizures	92	–
	Mean seizure frequency (per week)	–	24.99
	Participants reporting no seizures	80	
FND Type	Dissociative Seizures	98	41.9%
	Motor Symptoms	90	38.5%
	Functional Cognitive Symptoms	9	3.8%
	Functional Sensory Symptoms or Pain	9	3.8%
	Unknown/other	12	5.1%

‘Unknown/other’ encompasses participants for whom the FND subtype was not reported or clinically unclear.

At baseline, a substantial proportion of participants met established clinical cut-offs for caseness. 57% scored ≥10 on the PHQ-9, consistent with at least moderate depressive symptomatology (29). 46% scored ≥10 on the GAD-7, indicating at least moderate anxiety severity (28). 71% exceeded the clinical threshold on the CORE-10 (≥11), suggestive of clinically significant psychological distress (35). Additionally, 88% scored ≥10 on the WSAS, indicating at least moderate functional impairment (32).

184 participants provided pre-treatment data. 71 participants provided mid-treatment data and 53 provided end-treatment data. As participants provided data at one, two, or three timepoints, the number of responses included in each model ranged from 226 to 233 for each outcome measure. It was noted that after entering treatment, the number of sessions participants received inclusive of assessment varied widely from a single session, up to one patient who had sixty-one sessions. For some participants (n = 16), incorrect entry of their identification number meant the number of sessions they underwent was unknown. The mean number of sessions across all participants was 13.6.

### 3.1 Baseline analysis

Baseline statistics were calculated using all data provided at the pre-treatment timepoint. These are summarised in **Table 2**.

**Table 2:** Summary statistics for each patient reported outcome measure by timepoint.

Outcome	Pre	Mean (SD)	Clinical meaning	Mid	Mean (SD)	Clinical meaning	End	Mean (SD)	Clinical meaning
	N			N			N		
PHQ-9	180	13.37 (6.41)	Moderate depression (10–14)	69	10.27 (6.48)	Moderate depression	53	8.18 (6.02)	Mild depression
GAD-7	182	10.76 (6.26)	Moderate anxiety (10–14)	71	8.41 (6.08)	Mild anxiety	53	6.16 (4.98)	Mild anxiety
CORE-10	181	18.31 (8.12)	Moderate distress (15-19)	71	14.54 (8.73)	Moderate distress	53	11.42 (7.56)	Mild distress
Fear Avoidance Beliefs	181	9.23 (2.97)		71	8.45 (2.98)		53	7.83 (3.13)	
Damage Beliefs	180	9.78 (2.50)		71	8.20 (2.69)		53	7.83 (3.13)	
Embarrassment Avoidance Beliefs	180	10.93 (3.58)		69	10.46 (3.63)		53	9.40 (3.73)	
Symptom Focusing	182	11.09 (2.92)		71	9.92 (3.56)		53	9.53 (2.99)	
All-or-Nothing behaviour	180	10.74 (3.09)		70	10.27 (3.55)		53	9.47 (3.45)	
Avoidance–Resting behaviour	181	8.85 (3.25)		71	7.61 (3.34)		52	6.85 (2.88)	
WSAS	183	27.47 (10.47)	Moderately severe or worse functional impairment (≥20)	69	24.62 (11.90)	Moderately severe or worse functional impairment	53	20.49 (10.54)	Moderately severe or worse functional impairment
Physical Functioning (RAND-36)	176	57.42 (31.34)		71	62.93 (32.79)		53	67.74 (31.16)	
Bodily Pain (RAND-36)	176	46.78 (29.14)		68	54.41 (31.40)		53	56.27 (27.18)	

### 3.2 Mixed effects models

A summary of the twelve mixed effects models can be seen in **Table 3**. After correction, 9 of 12 outcomes showed a significant effect of Time.

**Table 3:** A summary of the twelve mixed effects models.

Outcome	Time effect	p (time)	FDR q<.05?	Estimated marginal means (SE)	Mean difference pre–end (95% CI)	Pairwise comparison p Pre-Mid	Pre-end	Mid-end
PHQ-9	F(2, 65.86) = 14.842	**<.001**	Yes	Pre 13.146 (.466) Mid 10.670 (.608) End 9.202 (.747)	-3.94 (-5.35, -2.40)	**<.001**	**<.001**	.071
GAD-7	F(2, 60.993) = 15.116	**<.001**	Yes	Pre 10.654 (.445) Mid 8.569 (.553) End 6.899 (.679)	-3.88 (-5.35, -2.40)	**<.001**	**<.001**	**.020**
CORE-10	F(2, 76.553) = 12.828	**<.001**	Yes	Pre 17.828 (.574) Mid 15.499 (.735) End 13.048 (1.026)	-4.78 (-6.95, -2.61)	**.001**	**<.001**	**.022**
Fear-Avoidance	F(2, 66.609) = 3.669	**.031**	Yes	Pre 9.190 (.213) Mid 8.453 (.312) End 8.445 (.323)	-0.75 (-1.44, -0.06)	**.025**	**.032**	.982
Damage Beliefs	F(2, 58.583) = 17.699	**<.001**	Yes	Pre 9.745 (.182) Mid 8.583 (.275) End 7.894 (.360)	-1.85 (-2.54, -1.15)	**<.001**	**<.001**	.089
Embarrassment-Avoidance	F(2, 53.269) = 2.159	.125	No	Pre 10.806 (.248) Mid 10.652 (.320) End 9.695 (.572)	-1.11 (-2.45, 0.23)	.282	.056	.171
Symptom Focus	F(2, 53.615) = 8.896	**<.001**	Yes	Pre 11.019 (.210) Mid 10.294 (.301) End 9.493 (.394)	-1.53 (-2.54, -0.52)	**.013**	**<.001**	.063
All-or-Nothing behaviour	F(2, 59.33) = 8.215	**<.001**	Yes	Pre 10.853 (0.222) Mid 10.287 (0.323) End 9.127 (0.430)	-1.73 (-2.77, -0.69)	.085	**<.001**	**.017**
Avoidance-Resting behaviour	F(2, 94.087) = 8.845	**<.001**	Yes	Pre 8.748 (.230) Mid 7.941 (.337) End 7.220 (.354)	-1.53 (-2.37, -0.69)	**.023**	**<.001**	.082
WSAS	F(2, 67.32) = 10.84	**<.001**	Yes	Pre 26.96 (.75) Mid 25.50 (1.06) End 21.82 (1.14)	-5.14 (-7.76, -2.52)	.144	**<.001**	**.004**
Physical Functioning (RAND PF)	F(2, 46.502) = 3.082	.055	No	Pre 58.551 (2.214) Mid 61.492 (2.580) End 64.347 (2.700)	+5.80 (-0.31, 11.92)	.173	**.016**	.205
Pain score (RAND Bodily Pain, 0–100)	F(2, 59.252) = 1.769	.179	No	Pre 47.906 (2.122) Mid 51.461 (3.083) End 53.288 (2.927)	+5.38 (-1.21, 11.97)	.250	.076	.592

Statistically significant p values are indicated in bold. PHQ-9, Patient Health Questionnaire-9; GAD-7, General Anxiety Disorder-7; CORE-10, Clinical Outcomes in Routine Evaluation; WSAS, Work and Social Adjustment Scale; RAND PF, physical functioning measure on the RAND 36-Item Health Survey; FDR, False Discovery Rate; SE, standard error.

#### 3.2.1 Mood and psychological distress

As seen in **Table 2**, depression (PHQ-9), anxiety (GAD-7), and general distress (CORE-10) all decreased significantly over time. The first linear mixed-effects model showed a significant effect of Time on PHQ-9 score. Pairwise (LSD, unadjusted) contrasts indicated a significant Pre-End and Pre-Mid decrease, while Mid-End showed a further decrease of 1.47 points that did not reach significance.

Similarly for the GAD-7 outcome measure, estimated marginal means decreased over time, with a significant overall effect of time. Pairwise (LSD, unadjusted) contrasts showed a significant difference in scores at the Pre-End, Pre-Mid and Mid-End timepoints. This was also seen for CORE-10 scores, which decreased significantly over time. Pairwise comparisons showed a significant Pre-End, Pre-Mid and Mid-End reduction in CORE-10 scores.

#### 3.2.2 Cognitive and behavioural responses

Mixed effects models were also run for each of the cognitive process outcomes: Fear-Avoidance beliefs, Damage Beliefs, Embarrassment-Avoidance beliefs, Symptom Focusing, and for All-or-Nothing thinking and Avoidance/Resting. Five of the six sub-scales showed a significant effect of Time, with reductions from Pre to End that remained significant after correction: Fear-Avoidance, Damage Beliefs, Symptom Focusing, All-or-Nothing thinking, and Avoidance/Rest (all q <.05). Embarrassment-Avoidance decreased numerically but was not statistically significant after correction.

#### 3.2.3 Physical functioning and pain

A linear fixed effects model showed a significant overall effect of Time on functional impairment score on the WSAS.

By contrast, the Physical Functioning scale trended toward improvement, but the overall Time effect was not statistically significant following FDR correction. The Pain score also showed a small, non-significant change over time.

### 3.3 Assessment of missing data

Notably, while 184 participants provided pre-treatment data, only 71 provided mid-treatment data and only 53 completed measures at the end-treatment timepoint. 176 participants provided data at only one timepoint, 42 provided data at exactly two timepoints, and 16 provided data for all three timepoints.

To assess whether the participants providing mid or post-treatment in addition to pre-treatment data differed systematically to those who only provided pre-treatment data, group comparisons were made. Independent samples t-tests for each of the twelve outcome measures, and none showed any significant difference between those providing data at pre-treatment only compared to those providing additional follow-up data (p <0.05).

### 3.4 Sensitivity analysis

In view of the wide range of sessions participants received, a sensitivity analysis was conducted after removal of data points (n = 33) provided by participants who received three or fewer treatment sessions, or for whom the number of treatment sessions was unknown. This left a total of 275 data points. The mixed-effects models were repeated using this reduced dataset. For every outcome that showed a significant effect of time in the full dataset (PHQ-9, GAD-7, Fear-Avoidance, Damage Beliefs, Symptom Focusing, All-or-Nothing behaviour, Avoidance/Resting, WSAS, and CORE10), the time effect also remained statistically significant in the reduced dataset. Similarly, outcomes that were not significant in the full dataset (physical functioning, pain, and embarrassment-avoidance) remained non-significant in the reduced dataset.

## 4 Discussion

Overall, the results show consistent improvement in psychological distress, everyday functioning as measured by the WSAS, and most targeted cognitive-behavioural processes across treatment, with no reliable change in pain and a trend towards improvement in the RAND-PF measure of physical functioning. This pattern of results remained unchanged after controlling for multiple testing using a Benjamini-Hochberg false discovery rate correction.

The observed reductions in the three psychological distress measures (PHQ-9, GAD-7 and CORE-10), indicating improvements in mood and reductions in distress over the course of treatment, are consistent with previous evaluations of CBT for FND and related presentations, including the CODES trial (23), multidisciplinary interventions (26) and systematic review findings (24). Similarly, improvements across five of six cognitive-behavioural response domains (Fear-Avoidance, Damage Beliefs, Symptom Focusing, All-or-Nothing behaviour, and Avoidance/Resting) support contemporary models that emphasise unhelpful beliefs, attentional focus, and avoidance behaviours as key mechanisms maintaining symptoms. Although we were not able to formally determine whether changes in cognitive and behavioural responses mediated improvements in other outcome measures, the observed pattern of change is consistent with mechanistic models of FND and with prior work suggesting that reductions in avoidance, maladaptive beliefs, and symptom-focused attention are central to therapeutic improvement (13, 19). This suggests that the CBT intervention may be associated with change in cognitive and behavioural processes believed to contribute to the maintenance of FND symptoms. These findings are therefore consistent with mechanistic models of FND, suggesting that CBT may influence cognitive and behavioural processes implicated in symptom maintenance; although it is noted that the present design does not allow causal conclusions regarding specific mechanistic pathways. Notably, improvements were observed across both cognitive domains (e.g., damage beliefs, symptom focusing) and behavioural domains (e.g., avoidance and activity patterns), which aligns with CBT models that emphasise reciprocal interactions between cognition and behaviour in maintaining functional symptoms. Prior CBT protocols for FND and related conditions have similarly reported that changes in maladaptive beliefs and avoidance behaviours are associated with improvements in distress and participation (36), even where changes in perceived physical capability are less pronounced (20, 23). Taken together, this pattern supports the interpretation that CBT may contribute to improvements in functioning and wellbeing through modification of cognitive-behavioural maintenance processes.

The dissociation observed between changes in psychological distress and changes in functioning is consistent with prior work in FND (23). In the present study, a significant improvement was observed on the WSAS, a global measure of functional impairment and participation across work, social, and domestic domains, whereas RAND-36 Physical Functioning and Pain subscales, which assessed perceived limitations in specific physical activities (e.g. walking, lifting, climbing stairs), did not show significant change over time. This suggests that the CBT intervention primarily reduced the extent to which difficulties interfered with everyday participation, rather than producing significant change in perceived physical capability. Such a profile of change aligns with process-focused CBT models that prioritise modification of transdiagnostic cognitive and behavioural mechanisms including attentional bias, threat appraisal, and avoidance, as described in secondary-care CBT programmes such as PRINCE (20). For many patients, improved mood, reduced psychological distress and alterations in thinking processes and coping mechanisms may constitute meaningful therapeutic benefit with wide-reaching implications for quality of life.

The overall pattern of results supports the potential benefit of CBT for those with FND, and suggests that CBT may help reduce distress, increase participation in life, and contribute to change in several key cognitive and behavioural maintenance processes. Clinically, these findings highlight the importance of psychoeducation that explicitly distinguishes between changes in wellbeing and participation and changes in perceived physical capability or symptom-related limitations. Helping patients to understand that improvements in distress and daily functioning may occur even when difficulties with physical activities persist may support more realistic expectations and sustained engagement with therapy, while also suggesting that CBT interventions may need to be modified or enhanced to more directly target perceived physical capability and activity-related limitations.

A substantial proportion of participants provided data at only one timepoint, with fewer contributing mid- or end-treatment assessments. Importantly, those who provided follow-up data did not differ at baseline from those who contributed only pre-treatment data, suggesting that missingness was unlikely to be driven by initial symptom severity or functioning. The mixed-effects modelling approach was selected to allow inclusion of all available data, minimising selection bias that may result from restricting analysis to complete cases. However, the high proportion of missing data nonetheless limits our ability to accurately describe clinical outcomes in the treatment group overall. This highlights the need to further improve data collection procedures within time-limited clinical settings; for example, integrating digital measures, automated reminders, or therapist-led completion of outcome measures during sessions. Recent qualitative work (37) provides important context for these challenges, highlighting stakeholder concerns regarding the relevance, burden, and timing of commonly used outcome measures in FND. Participants emphasised the importance of capturing changes in coping, understanding, participation, and quality-of-life, and identified measurement burden as a barrier to consistent completion, highlighting the need for outcome frameworks and data collection procedures that are both clinically meaningful and feasible in routine care.

In addition to missing data, this study has several limitations. Firstly, the study made retrospective use of routinely collected clinical outcomes data and did not feature randomisation or make comparisons to a control group. This therefore prevents conclusions about causality. The findings reported could be due to random symptom fluctuation within this cohort. The lack of a control group also means that it is not possible to determine whether the improvements in outcome measures were the result of the active therapeutic components of the CBT intervention as opposed to placebo effect, or the non-specific influence of a supportive therapeutic alliance. The relatively small sample size also limits the extent to which findings can be generalised to the broader FND population. Our sample was comprised of predominantly female, White British participants. Although this broadly reflects the population of those diagnosed with FND (38, 39), it limits generalisability to those of other racial and/or gender identities and highlights the ongoing need for inclusive research and service evaluation strategies. The sample was also predominantly composed of individuals with motor FND and functional seizures. Whilst this appears to reflect typical specialist service referrals, caution is warranted when generalising to other FND presentations. Sensory presentations were recorded as a single category in the clinical dataset; given the heterogeneity within sensory FND, this grouping may obscure subtype-specific differences and limits the precision of conclusions for these presentations. Limited information was available regarding participants’ comorbid psychiatric diagnoses, symptom severity, and concurrent treatments, including medication and other psychological interventions. Although baseline analysis of caseness indicated that a substantial proportion of participants met established thresholds for clinically significant depression, anxiety, psychological distress, and functional impairment, more detailed diagnostic characterisation (e.g., formal psychiatric diagnoses) was not consistently available within the routinely collected dataset. These factors may have influenced treatment outcomes and represent potential confounding variables that could not be controlled for in the present analysis.

Furthermore, although the study was conceptually grounded in a biopsychosocial framework, outcome measurement primarily focused on psychological and behavioural process-level mechanisms and functional impairment. Social, relational, and environmental influences, which are also recognised as important contributors to symptom maintenance and recovery in FND, were not directly assessed. Future research incorporating measures of interpersonal functioning, occupational context, and broader environmental factors may help to more comprehensively evaluate the contribution of biopsychosocial processes to treatment outcomes.

Despite these limitations, the findings indicate that CBT may offer meaningful benefits for people with FND by reducing distress, improving functional impairment, and modifying unhelpful cognitive and behavioural responses for those with FND. Clinically, these results suggest a need to consider how CBT interventions might be adapted, enhanced, or combined with additional therapeutic approaches to more directly target outcomes such as perceived physical functioning and pain, where these remain unchanged. Clarifying that such changes may occur even in the absence of improvements in physical functioning or pain could assist clinicians in psychoeducation and in establishing accurate expectations and treatment goals, thereby supporting more collaborative and informed therapeutic engagement.

To conclude, this study provides evidence that CBT for FND is associated with significant improvements in mood, distress, functional impairment, and several key cognitive and behavioural processes implicated in symptom maintenance. Consistent with existing findings in the field, there was no significant change in physical functioning and pain. Future studies could make use of more recent developments in the recommended outcome measurement priorities to ensure reliable routine outcome monitoring, as well as using larger-scale studies (including non-seizure FND groups) to examine the psychological mechanisms by which CBT interventions bring about change. This is hoped to complement other psychological approaches currently being explored in FND, including EMDR (40) and acceptance and commitment therapy (ACT) (41), both as standalone treatments and in comparison or combination with CBT.

## Data Availability

The original contributions presented in the study are included in the article/supplementary material. Further inquiries can be directed to the corresponding author/s.

## References

[1] American Psychological Association . Diagnostic and statistical manual of mental disorders, 5th ed. (2013).

[2] Drane DL Fani N Hallett M Khalsa SS Perez DL Roberts NA . A framework for understanding the pathophysiology of functional neurological disorder. CNS spectrums. (2020) 26. doi: 10.1017/S1092852920001789, PMID: 32883381 PMC7930164

[3] Stone J Carson A Duncan R Roberts R Warlow C Hibberd C . Who is referred to neurology clinics? The diagnoses made in 3781 new patients. Clin Neurol Neurosurg. (2010) 112. doi: 10.1016/j.clineuro.2010.05.011, PMID: 20646830

[4] Lidstone SC Costa-Parke M Robinson EJ Ercoli T Stone J . Functional movement disorder gender, age and phenotype study: a systematic review and individual patient meta-analysis of 4905 cases. J Neurology Neurosurg Psychiatry. (2022) 93. doi: 10.1136/jnnp-2021-328462, PMID: 35217516

[5] McLoughlin C Hoeritzauer I Cabreira V Aybek S Adams C Alty J . Functional neurological disorder is a feminist issue. J Neurology Neurosurgery Psychiatry. (2023) 94. doi: 10.1136/jnnp-2022-330192, PMID: 36977553 PMC10511956

[6] Fox J Humayun MJ Bollig MK Hopkins BW Mishra M . Trauma and health-related quality of life in patients with functional seizures. Seizure: Eur J Epilepsy. (2025) 131. doi: 10.1016/j.seizure.2025.08.009, PMID: 40803196 PMC13237828

[7] Pick S Goldstein LH Perez DL Nicholson TR . Emotional processing in Functional Neurological Disorder: A review, biopsychosocial model and research agenda. J neurology neurosurgery Psychiatry. (2019) 90. doi: 10.1136/jnnp-2018-319201, PMID: 30455406 PMC6525039

[8] Edwards M Adams RA Brown H Pareés I Friston KJ . A bayesian account of ‘hysteria’. Brain: J Neurol. (2012) 135. doi: 10.1093/brain/aws129, PMID: 22641838 PMC3501967

[9] Huys AML Bhatia KP Haggard P Edwards MJ . Symptom-triggered attention to self as a possible trigger of functional comorbidity. Mov Disord Clin Pract. (2021) 8:159–61. doi: 10.1002/mdc3.13120, PMID: 33426174 PMC7781079

[10] Perez DL Nicholson TR Asadi-Pooya AA Bègue I Butler M Carson AJ . Neuroimaging in functional neurological disorder: state of the field and research agenda. NeuroImage. Clin. (2021) 30:102623. doi: 10.1016/j.nicl.2021.102623, PMID: 34215138 PMC8111317

[11] Ludwig L Pasman JA Nicholson T Aybek S David AS Tuck S . Stressful life events and maltreatment in conversion (functional neurological) disorder: systematic review and meta-analysis of case-control studies. Lancet Psychiatry. (2018) 5:307–20. doi: 10.1016/S2215-0366(18)30051-8, PMID: 29526521

[12] Civilotti C Nistico V Tedesco R Cuoco S Bisogno R Barone P . Attachment State of Mind and complex traumatization in patients with Functional Motor Disorder (Motor Conversion Disorder). Brain Behav Immun Health. (2024) 42:100913. doi: 10.1016/j.bbih.2024.100913, PMID: 39655281 PMC11626536

[13] Brown RJ Reuber M . Towards an integrative theory of psychogenic non-epileptic seizures (PNES). Clin Psychol Rev. (2016) 47:55–70. doi: 10.1016/j.cpr.2016.06.003, PMID: 27340856

[14] Chalder T . Cognitive behavioural therapy as a treatment for conversion disorders. In: Contemporary approaches to the study of hysteria. Oxford Academic, New York, NY (2021).

[15] Chalder T . Non-epileptic attacks: A cognitive behavioural approach in a single case with a four-year follow-up. Clin Psychol Psychother. (1996) 3. doi: 10.1002/(SICI)1099-0879(199612)3:4<291::AID-CPP72>3.3.CO;2-8

[16] Moro P Lattanzi S Beier CP Di Bonaventura C Irelli EC . Cognitive behavioral therapy in adults with functional seizures: A systematic review and meta-analysis of randomized controlled trials. Epilepsy Behav. (2025) 159. doi: 10.1016/j.yebeh.2024.109981, PMID: 39181107

[17] Mavroudis I Petrides F Franekova K Ionescu C Ciobica A Kazis D . Treatment of functional neurological disorder: an umbrella review of systematic reviews and meta-analyses. J Neurol. (2025) 272:710. doi: 10.1007/s00415-025-13449-7, PMID: 41111084

[18] James K Patel M Goldsmith K Moss-Morris R Ashworth M Landau S . Transdiagnostic therapy for persistent physical symptoms: A mediation analysis of the PRINCE secondary trial. Behav Res Ther. (2022) 159. doi: 10.1016/j.brat.2022.104224, PMID: 36379081

[19] Chalder T Landau S Stone J Carson A Reuber M Medford N . How does cognitive behavior therapy for dissociative seizures work? A mediation analysis of the CODES trial. psychol Med. (2024) 54. doi: 10.1017/S0033291723003665, PMID: 38197148

[20] Chalder T Patel M James K Hotopf M Frank P Watts K . Persistent physical symptoms reduction intervention: a system change and evaluation in secondary care (PRINCE secondary) - a CBT-based transdiagnostic approach: study protocol for a randomised controlled trial. BMC Psychiatry. (2019) 19. doi: 10.1186/s12888-019-2297-y, PMID: 31640632 PMC6805658

[21] Hofmann SG Hayes SC . The future of intervention science: process-based therapy. Clin Psychol Sci. (2019) 7:37–50. doi: 10.1177/2167702618772296, PMID: 30713811 PMC6350520

[22] Goldstein L Chalder T Chigwedere C Khondoker M Moriarty J Toone B . Cognitive-behavioral therapy for psychogenic nonepileptic seizures: A pilot RCT. Neurology. (2010) 74. doi: 10.1212/WNL.0b013e3181e39658, PMID: 20548043 PMC2905892

[23] Goldstein LH Robinson EJ Mellers JDC Stone J Carson A Reuber M . Cognitive behavioural therapy for adults with dissociative seizures (CODES): a pragmatic, multicentre, randomised controlled trial. Lancet Psychiatry. (2020) 7. doi: 10.1016/S2215-0366(20)30128-0, PMID: 32445688 PMC7242906

[24] Gutkin M McLean L Brown R Kanaan RA . Systematic review of psychotherapy for adults with functional neurological disorder. J Neurology Neurosurg Psychiatry. (2021) 92. doi: 10.1136/jnnp-2019-321926, PMID: 33154184

[25] Gaskell C Power N Novakova B Simmonds-Buckley M Kerr WT Reuber M . A meta-analytic evaluation of the effectiveness and durability of psychotherapy for adults presenting with functional dissociative seizures. Seizure - Eur J Epilepsy. (2024) 119. doi: 10.1016/j.seizure.2024.05.016, PMID: 38824867

[26] Guy L Caceres G Jackson T Gorman S Wilson J Hsieh Y . Routine outcomes and evaluation of an 8-week outpatient multidisciplinary rehabilitative therapy program for functional neurological disorder. J Neurol. (2024) 271. doi: 10.1007/s00415-023-12111-4, PMID: 38091087 PMC10973040

[27] Pick S Anderson DG Asadi-Pooya AA Aybek S Baslet G Bloem BR . Outcome measurement in functional neurological disorder: a systematic review and recommendations. J Neurology Neurosurg Psychiatry. (2020) 91. doi: 10.1136/jnnp-2019-322180, PMID: 32111637 PMC7279198

[28] Spitzer RL Kroenke K Williams JBW Löwe B . A brief measure for assessing generalized anxiety disorder. Arch Internal Med. (2006) 166. doi: 10.1001/archinte.166.10.1092, PMID: 16717171

[29] Kroenke K Spitzer RL Williams JBW . The PHQ-9: validity of a brief depression severity measure. J Gen Internal Med. (2001) 16. doi: 10.1046/j.1525-1497.2001.016009606.x, PMID: 11556941 PMC1495268

[30] Rosenström TH Mylläri S Malkki V Saarni SE . Feasibility of generic, short, and easy-to-use assessment of psychological distress during psychotherapy: Longitudinal measurement invariance of CORE-10 and -OM. Psychother Res. (2022) 32. doi: 10.1080/10503307.2022.2074807, PMID: 35580272

[31] Hays R Sherbourne C Mazel R . The RAND 36-item health survey 1.0. Health Econ. (1993) 2. doi: 10.1037/t08623-000, PMID: 8275167

[32] Mundt J Marks I Shear M Greist J . The Work and Social Adjustment Scale: a simple measure of impairment in functioning. Br J psychiatry: J Ment Sci. (2002) 180. doi: 10.1192/bjp.180.5.461, PMID: 11983645

[33] Ryan E Vitoratou S Goldsmith K Chalder T . Psychometric properties and factor structure of a long and shortened version of the cognitive and behavioural responses questionnaire. Psychosomatic Med. (2018) 80. doi: 10.1097/PSY.0000000000000536, PMID: 29023262

[34] Picariello F Chilcot J Chalder T Herdman D Moss-Morris R . The Cognitive and Behavioural Responses to Symptoms Questionnaire (CBRQ): Development, reliability and validity across several long-term conditions. Br J Health Psychol. (2023) 28. doi: 10.1111/bjhp.12644, PMID: 36690909

[35] Connell J Barkham M . CORE-10 user manual, version 1.1. CORE System Trust & CORE Information Management Systems Ltd, Rugby, UK. (2007).

[36] LaFrance WC Baird GL Barry JJ Blum AS Frank Webb A Keitner GI . Multicenter pilot treatment trial for psychogenic nonepileptic seizures: a randomized clinical trial. JAMA Psychiatry. (2014) 71:997–1005. doi: 10.1001/jamapsychiatry.2014.817, PMID: 24989152

[37] Rutten S Bradley-Westguard A Nicholson TR Pick S . Outcome measurement in functional neurological disorder: A qualitative study on the views of patients, caregivers and healthcare professionals. J Neurol. (2025) 272. doi: 10.1007/s00415-025-12912-9, PMID: 39932584 PMC11814038

[38] Hallett M Aybek S Dworetzky BA McWhirter L Staab J Stone J . Functional neurological disorder: new phenotypes, common mechanisms. Lancet Neurol. (2022) 21. doi: 10.1016/S1474-4422(21)00422-1, PMID: 35430029 PMC9107510

[39] O’Mahony BW Nelson-Sice R Nielsen G Hunter R Cope S Agarwal N . Cross-sectional evaluation of health resource use in patients with functional neurological disorders referred to a tertiary neuroscience centre. BMJ Neurol Open. (2024) 6. doi: 10.1136/bmjno-2023-000606, PMID: 38800070 PMC11116875

[40] Cope SR Smith JG El-Leithy S Vanzan S Hogwood P Golder D . Randomised feasibility study evaluating eye movement desensitisation and reprocessing therapy for functional neurological disorder (MODIFI). J Neurol. (2025) 272. doi: 10.1007/s00415-025-13219-5, PMID: 40627223 PMC12238153

[41] Poole N Cope S Vanzan S Duffus A Mantovani N Smith J . Feasibility randomised controlled trial of online group Acceptance and Commitment Therapy for Functional Cognitive Disorder (ACT4FCD). BMJ Open. (2023) 13. doi: 10.1136/bmjopen-2023-072366, PMID: 37169496 PMC10186420

